# Delivering the right care to people with low back pain in low- and middle-income countries: the case of Nepal

**DOI:** 10.7189/jogh.09.010304

**Published:** 2019-06

**Authors:** Sweekriti Sharma, Adrian C Traeger, Shiva Raj Mishra, Saurab Sharma, Chris G Maher

**Affiliations:** 1 Sydney School of Public Health, Faculty of Medicine and Health, The University of Sydney, Sydney, Australia; 2 Institute for Musculoskeletal Health, Sydney, Australia; 3 Nepal Development Society, Bharatpur, Nepal; 4 Kathmandu University School of Medical Sciences, Dhulikhel, Nepal

A recent *Lancet* series [1] and World Health Organization commission [2] have highlighted the urgent need for all nations to increase attention on delivering appropriate, affordable care for non-communicable diseases (NCDs). One of the most critically under-prioritised NCDs in Nepal is low back pain.

Globally, 60.1 million disability-adjusted life-years were due to low back pain in 2015, which increased by 54% since 1990 [3]. The most notable increase was in low- and middle-income countries. According to Global Burden of Disease data, low back pain is the number one cause of disability in Nepal; since 2005 the number of years lived with disability due to low back pain in Nepal increased by 16.9% [4]. Nepal has a largely agrarian economy and one quarter of the population live below the poverty line. Inability to perform physically demanding work due to low back pain will drive many Nepali people into poverty. High health care cost alone could drive an additional 800 000 people into poverty each year [5].

Nepal faces major challenges to reduce the burden of low back pain. There is an undersupply of health professionals with adequate training [6]. Nepal has less than one physiotherapist per 20 000 people [7], most of whom work in urban areas (Australia has 24 physiotherapists per 20 000 people). Moreover, the *Lancet* series [1] identified a worrying trend: health professionals in low- and middle-income countries providing the wrong care for low back pain. Over a three-month period in 2012, Nepali doctors ordered 722 MRIs to investigate the cause of low back pain [8]. MRIs have little clinical value for low back pain and would cost the average Nepali person approximately one third of their monthly household income. Although Nepal began piloting of insurance schemes as early as 1990s, they are yet to achieve tangible results. Furthermore, only 6.4% of the total government health spending was allocated to NCDs in 2014 [6].

Resource-poor settings such as Nepal have a tremendous task ahead of them. It is unrealistic to expect governments to shift spending away from urgent issues such as maternal health (only 58% of deliveries in Nepal had skilled birth attendants present) to non-life-threatening conditions such as low back pain. Given the profound socioeconomic burden of low back pain, a wise place to start would be to integrate care for low back pain with existing services. South Africa recently integrated back pain information into a national NCD project which spanned community, work and school settings [9]. Community-led programs for peer-education targeting physical activity [10] could also be a useful entry point for integrating information about low back pain. This approach would accord with the World Health Organization’s ‘best buy’ interventions – cost-effective interventions to improve health for ≤I$ 100 per DALY averted. These strategies should be accompanied by increased investment in affordable, evidence-based services for low back pain.

The Nepal NCD and Injury Poverty Commission listed 25 priority NCDs and injuries. Low back pain was not one of them [6]. With 2018 being the year to address NCDs [2], we believe now is the time to prioritise low back pain in Nepal and other low- and middle-income countries.

**Figure:**
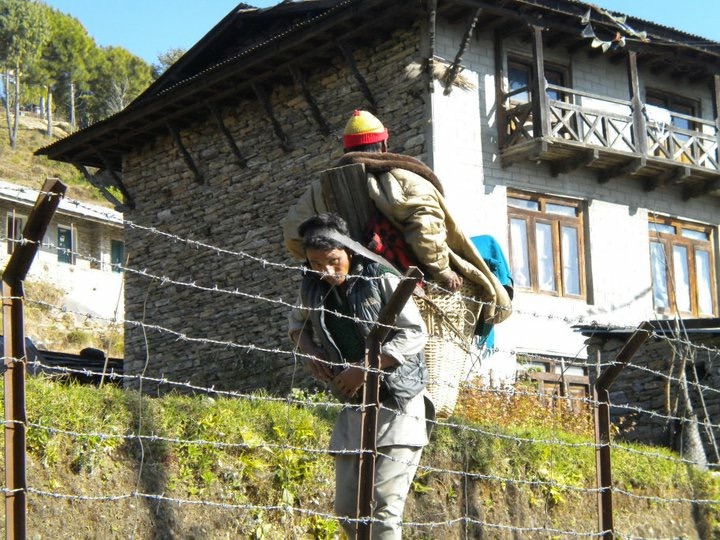
Photo: Patient being transported to the local health post (from the collection of Saurab Sharma, used with permission)
